# Intrafamily heterooligomerization as an emerging mechanism of methyltransferase regulation

**DOI:** 10.1186/s13072-024-00530-0

**Published:** 2024-03-01

**Authors:** Haley V. Hobble, Christine E. Schaner Tooley

**Affiliations:** Department of Biochemistry, Jacobs School of Medicine and Biomedical Sciences, State University of New York at Buffalo, Buffalo, NY 14203 USA

**Keywords:** Methyltransferase, Methylation, Oligomerization, Regulation

## Abstract

Protein and nucleic acid methylation are important biochemical modifications. In addition to their well-established roles in gene regulation, they also regulate cell signaling, metabolism, and translation. Despite this high biological relevance, little is known about the general regulation of methyltransferase function. Methyltransferases are divided into superfamilies based on structural similarities and further classified into smaller families based on sequence/domain/target similarity. While members within superfamilies differ in substrate specificity, their structurally similar active sites indicate a potential for shared modes of regulation. Growing evidence from one superfamily suggests a common regulatory mode may be through heterooligomerization with other family members. Here, we describe examples of methyltransferase regulation through intrafamily heterooligomerization and discuss how this can be exploited for therapeutic use.

## Introduction

The field of biological methylation has historically been driven by the well-characterized roles of DNA and histone methylation in transcriptional regulation [1, 2]. However, since their discovery, new protein and nucleic acid targets of methylation have been identified, and this modification is now known to also play key roles in cell signaling, metabolism, and translation [3–6]. This places methyltransferases as important players, not only in cancer progression, but also in metabolic and degenerative disorders and stem cell development [7–10].

Though *S*-adenosyl-methionine (SAM)-dependent methyltransferases have a wide variety of potential substrates, including DNA, RNA, proteins, lipids, and small molecules, they can be divided into a relatively small amount of superfamilies based on structural similarities [11]. The largest superfamily is the seven-β-strand (7βS/Class I) family, whose members share a conserved Rossmann fold-like structural core and include the PRMT (protein arginine methyltransferase), the DNMT (DNA methyltransferase), and the METTL (methyltransferase-like) families [11–14]. Many protein lysine methyltransferases are from the SET-domain (Su(var)3–9, Enhancer of zeste, Trithorax) superfamily, which is the second largest [11, 15]. In addition, there is the SPOUT superfamily, consisting primarily of RNA methyltransferases, and the smaller radical SAM, MetH activation, homocysteine, membrane, precorrin-like, and TYW3 superfamilies [11].

Despite the diverse substrate specificity that can be found within members of each superfamily, their structurally conserved active sites suggest superfamilies may also share modes of regulation. Growing evidence from the 7βS superfamily suggests one of these shared modes of regulation is heterooligomerization with close family members. Here, we describe examples of methyltransferase intrafamily heterooligomerization from three families within the 7βS superfamily (DNMT, PRMT, and METTL), highlight the regulatory roles of these interactions, and discuss how they can be targeted for therapeutic use.

## DNA methyltransferases (DNMT3A/DNMT3B/DNMT3L)

**Fig. 1:**
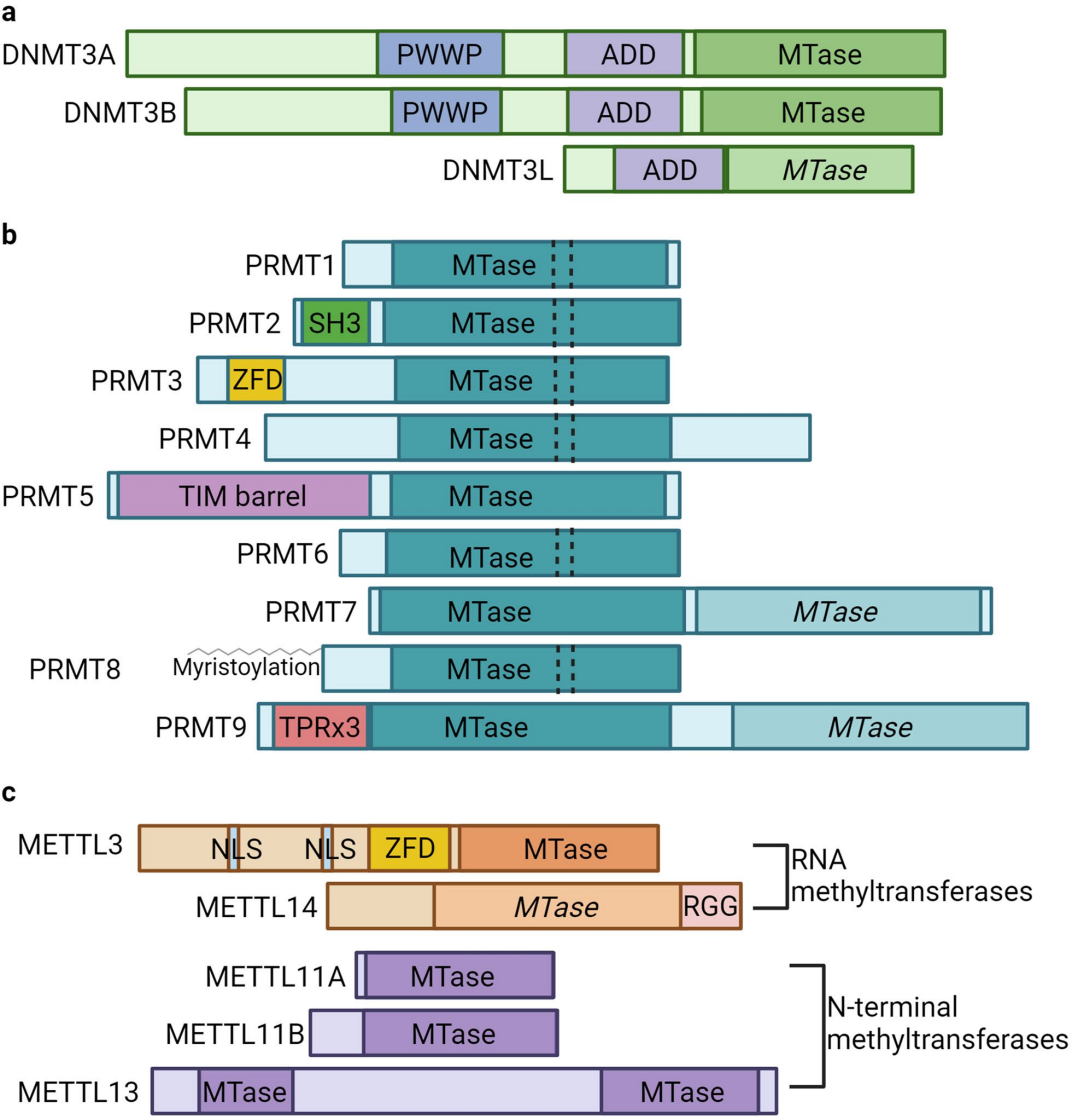
Domain architecture of methyltransferase families. **a** The de novo DNA methyltransferases, DNMT3A, DNMT3B, and DNMT3L all share a similar ADD (Atrx-Dnmt3-Dnmt3l) domain. DNMT3A and DNMT3B share a PWWP (Pro-Trp-Trp-Pro) domain and a conserved catalytic methyltransferase (MTase) domain. DNMT3L possesses an inactive MTase domain variant, shown in a lighter color and italicized to distinguish it from the active MTase domains. **b** The PRMT family consists of 9 enzymes containing a conserved catalytic MTase core. Several notable features are found at the N-termini of some PRMTs: PRMT2 contains an SH3 domain, PRMT3 contains a zinc finger domain (ZFD), PRMT5 contains a TIM barrel, PRMT8 is N-terminally myristoylated, and PRMT9 contains three TRP (tetratricopeptide) motifs. PRMT7 and PRMT9 contain two tandem MTase domains, with the C-terminal MTase domains lacking some of the conserved motifs of the canonical PRMT core rendering them catalytically inactive. The inactive MTase domains are italicized and shown in a lighter color than the active. Type I PRMTs also have a conserved dimerization arm shown with dashed lines within the MTase domains. **c** The METTL family members discussed here include the RNA (m6A) methyltransferases METTL3 and METTL14, and the N-terminal methyltransferases METTL11A, METTL11B, and METTL13. The m6A methyltransferase METTL3 contains two nuclear localization signals (NLS), and a ZFD at its N-terminus, while METTL14 possesses a catalytically inactive MTase domain (italicized and shown in a lighter color than the active MTase domain) and RGG repeats (RGG) at its C-terminus. The N-terminal methyltransferases METTL11A and METTL11B each only contain one active MTase domain, while METTL13 contains two active MTase domains with distinct functions. Created with Biorender.com

DNA methylation is an epigenetic modification important for several factors in mammalian development including genomic imprinting, X chromosome inactivation, and silencing of retrotransposons [16]. The major de novo DNA methyltransferases are DNMT3A and DNMT3B, and the major maintenance methyltransferase is DNMT1 [17]. Although DNMT3A and DNMT3B share several structural similarities, including closely related ADD (Atrx-Dnmt3-Dnmt3l) domains, PWWP (Pro-Trp-Trp-Pro) domains, and a highly conserved methyltransferase (MTase) catalytic domain (Fig. 1a), each enzyme possesses distinct functions and has different effects on development [18, 19]. DNMT3A is required for establishment of both maternal and paternal imprinting, and DNMT3B specifically methylates pericentromeric satellite repeats [18, 20]. Conformational differences in their catalytic loops can account for observed differences in substrate specificity between the two [21]. The third member of the DNMT3 family, DNMT3L, contains an ADD domain and some of the conserved catalytic motifs of DNMT3A and DNMT3B (Fig. 1a), but lacks critical amino acids within the MTase catalytic site, rendering it catalytically inactive [22]. Accordingly, crystallization studies have shown that only DNMT3A and DNMT3B, but not DNMT3L, can bind *S*-adenosyl-homocysteine (SAH) [21, 23–25].

**Fig. 2:**
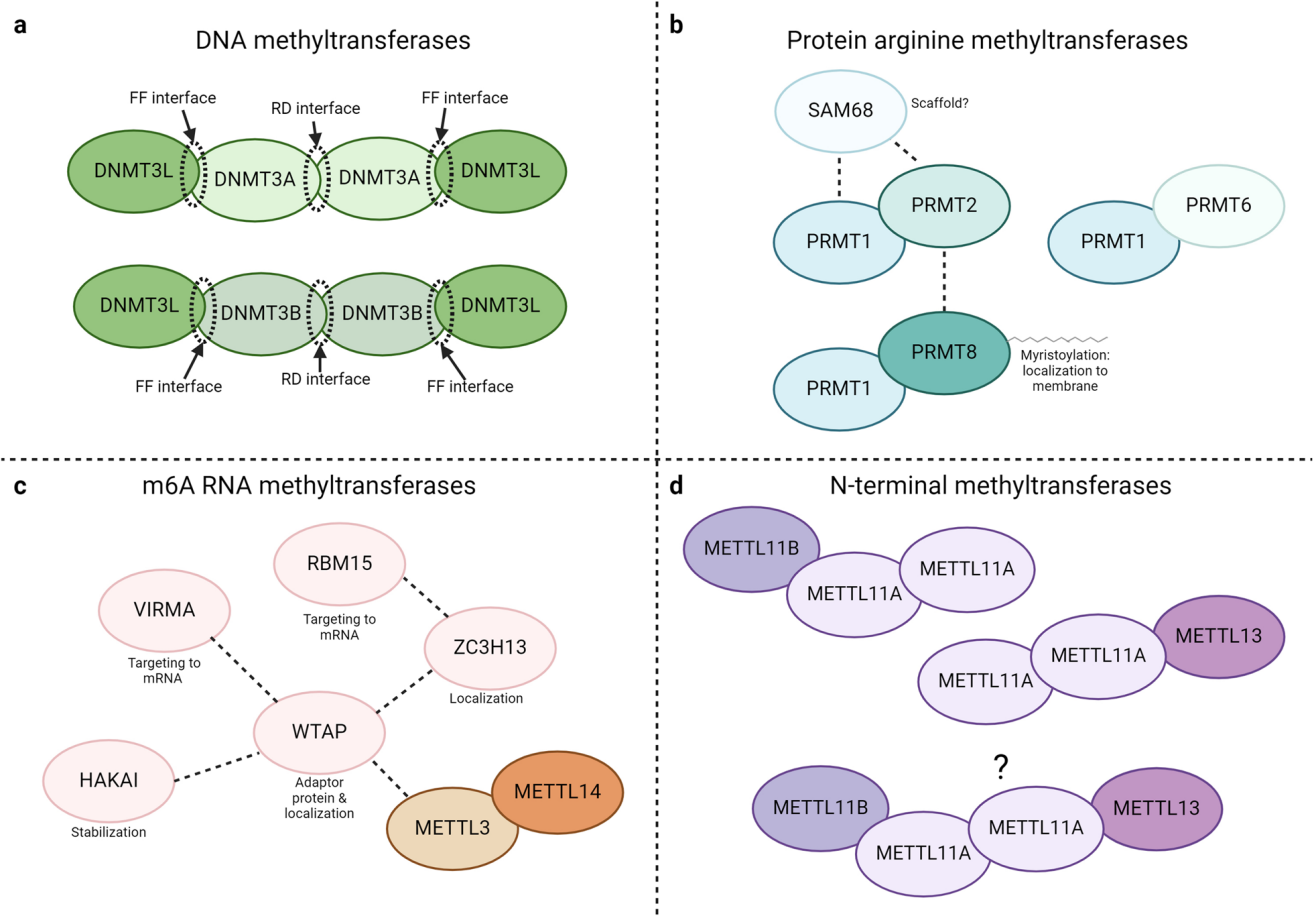
Heterooligomeric methyltransferase complex formation. **a** DNMT3A–DNMT3L or DNMT3B–DNMT3L heterotetramers are depicted with the positions of the outer FF interfaces and inner RD interfaces shown. These interfaces would be in the same locations for both types of homotetramers as well. **b** The PRMT1–PRMT6, PRMT1–PRMT2, and PRMT1–PRMT8 heterodimers are shown along with dashed lines to indicate additional interactions between PRMT2 and PRMT8, and PRMT1, PRMT2, and SAM68 (KH domain-containing, RNA-binding, signal transduction-associated protein 1). **c** The METTL3–METTL14 heterodimer is shown along with dashed lines indicating interactions with other members of the m6A methyltransferase complex. *WTAP* Wilms Tumor 1-Associating Protein, *VIRMA* Vir Like m6A Methyltransferase Associated, *HAKAI* E3 ubiquitin-protein ligase, *RBM15* RNA binding motif protein 15, *ZC3H13* Zinc Finger CCCH-Type Containing 13. **d** The METTL11A dimer is shown as a complex with METTL11B or METTL13 individually and in combination as one possible larger complex consisting of all three N-terminal methyltransferases. Created with BioRender.com

DNMT3A and DNMT3B can homodimerize, and two of these dimers can bind to form a linear homotetramer [26–28]. DNMT3A and DNMT3B can also heterodimerize with DNMT3L, and two of these heterodimers can bind to form a linear heterotetramer in a DNMT3L–DNMT3A/B–DNMT3A/B–DNMT3L arrangement (Fig. 2a) [21, 24]. In these tetramers, the outer interfaces between the original monomers are referred to as the FF interfaces, and the inner interface between the two dimers is called the RD interface (Fig. 2a) [21, 29]. In heterotetramers, the homodimeric DNMT3A–DNMT3A or DNMT3B–DNMT3B RD interface mediates DNA substrate binding [21, 23, 24]. DNMT3L does not make direct contact with the DNA, but instead the heterodimeric DNMT3L–DNMT3A/B FF interfaces, primarily formed by hydrophobic interactions, stabilize the DNA-binding conformations of DNMT3A or DNMT3B and enhance their methylation activities [21, 23, 24, 27]. In the case of DNMT3A, homotetramer formation helps promote its assembly into protein filaments, which allow simultaneous binding to two DNA molecules oriented in parallel [30]. It is thought the enhanced DNA-binding property of the DNMT3A filaments helps target the enzyme to DNA-dense heterochromatin regions [30]. However, heterotetramer formation with DNMT3L prevents DNMT3A from forming protein filaments, as the DNMT3L on the ends of the tetramer cannot further dimerize, and releases DNMT3A from heterochromatin [30]. In these ways, the catalytically inactive DNMT3L plays an important regulatory role for its active family members, DNMT3A and DNMT3B.

Though most DNA methyltransferases were originally thought to function as monomers, the discovery of the homotetramerization of DNMT3A demonstrated a new mode of methyltransferase regulation that promotes its assembly into protein filaments and reduces the rate of dissociation of DNMT3A from heterochromatin [17, 26, 30, 31]. The additional discovery that DNMT3A and DNMT3B can also heterotetramerize with DNMT3L further expanded our knowledge of methyltransferase regulation, illuminating a new way to differentially alter filament formation and sub-nuclear localization [17, 30, 32]. Together, the interactions occurring within the DNMT3 family demonstrate that heterooligomerization, along with homooligomerization, is a viable regulatory mechanism between methyltransferases within the same family, and that these different types of complexes have unique functions within the cell.

## Protein arginine methyltransferases (PRMTs)

Another family of methyltransferases that were first thought to only homooligomerize is the mammalian protein arginine methyltransferase (PRMT) family. Mammalian PRMTs can be divided into three different groups depending on the type of arginine modification that they place. Type I PRMTs (1, 2, 3, 4, 6, and 8) catalyze mono- and asymmetric dimethylation. Type II PRMTs (5 and 9) catalyze mono- and symmetric dimethylation, and the type III PRMT, PRMT7, catalyzes only monomethylation [33]. Members of the PRMT family share a canonical MTase catalytic domain consisting of a Rossmann fold, which encompasses the SAM-binding domain (Fig. 1b) [34, 35]. They also have variable N-terminal domains that can encompass an SH3 domain (PRMT2), a zinc finger domain (ZFD) (PRMT3), a TIM barrel (PRMT5), TPR repeats (PRMT9), or sites for myristoylation (PRMT8) (Fig. 1b) [34, 36–38]. Uniquely, type I PRMTs also contain a conserved dimeric interface, suggesting all but PRMT5, PRMT7, and PRMT9 function in a dimeric state (Fig. 1b) [34].

Homodimerization appears to be required for normal catalytic activity in Type I PRMTs, as dimerization-disrupting mutations found in both PRMT1 and PRMT3 diminish SAM-binding ability and catalytic activity [39]. Biochemically, the dimerization arm is very hydrophobic, so it is thought that homodimerization covers these patches and promotes stability [40]. Functionally, it has been hypothesized that homodimers form to engage residues on the other side of the structure in SAM binding or allow processive production of asymmetric dimethylarginine, with the first product, monomethylarginine, directly entering the second active site to become dimethylated [39].

However, some Type I PRMTs can also heterodimerize with other family members as a regulatory mechanism. In *Trypanosoma brucei*, the active PRMT1^ENZ^ heterodimerizes with an inactive paralog, PRMT1^PRO^ [41]. Though a prozyme with no catalytic activity of its own, PRMT1^PRO^ is required for PRMT1^ENZ^ activity because PRMT1^ENZ^ cannot homodimerize and is unstable on its own [41, 42]. PRMT1^PRO^ and PRMT1^ENZ^ form an antiparallel heterodimer that corresponds to the canonical homodimer of other PRMTs, and similar to DNMT3A/B and DNMT3L, two heterodimers complex to form a tetramer, with this tetramerization required for catalytic function [42]. The N-terminus of PRMT1^PRO^ is also the main driver of substrate binding, as deletion of this domain abolishes the affinity of the heterotetramer for substrate, even in the presence of WT PRMT1^ENZ^ [42].

Though in mammals PRMT1 is able to homodimerize [39], it also heterodimerizes with PRMT6, PRMT2, and PRMT8 [38, 43, 44]. Interaction between PRMT1 and PRMT6 allows PRMT1 to methylate PRMT6 and inhibit its methylation of histone H3 [44]. This is thought to promote a dual mechanism for PRMT1 to activate transcription, first by placing its own activating methylation of histone H4, and second, by inhibiting the repressive methylation of PRMT6 [44]. In contrast, PRMT2 is not known to be a substrate of PRMT1, but its binding activates PRMT1 by increasing both its *V*_max_ and substrate affinity [43]. Interestingly, the catalytic activity of PRMT2 is not needed for this activation, but instead the ability of PRMT2 to bind and activate PRMT1 is dependent on both the activity of PRMT1 and the presence of the PRMT2 SH3 domain, giving rise to the hypothesis that methylation of a different substrate(s) provides a scaffold for this interaction [43]. One candidate for such a scaffolding protein is the PRMT1 substrate, SAM68, which can interact with PRMT2 (Fig. 2b) and has previously been shown to mediate the interaction between PRMT1 and mixed lineage leukemia (MLL) methyltransferase through the MLL SH3 domain [43, 45, 46]. Though PRMT2 catalytic activity is not required for its activation of PRMT1, the PRMT1–PRMT2 heterodimer is unique from the DNMT3A/B-DNMT3L heterodimer in that PRMT2 does contain the conserved PRMT core motifs and has defined catalytic activity of its own [47]. This suggests that PRMT2 has both catalytic and non-catalytic roles.

As PRMT1 is the predominant human arginine methyltransferase and alone methylates over 85% of arginine substrates [48], the need for its heterodimerization remains unclear [43]. However, while it can serve all the same purposes as homodimerization, heterodimerization can also provide a variety of unique substrate preferences and/or localization patterns that enhance the diversity of PRMT1 functions [43]. Studying the PRMT1–PRMT8 heterodimer may give more insight into this. While the implications of the PRMT1–PRMT8 heterodimer are not yet understood [38], PRMT8 has tissue- and cell-specific expression, with high expression in neurons of the central nervous system, and is also the only PRMT to localize to the plasma membrane (mediated by its unique N-terminal myristoylation) [38]. It is possible that heterodimer formation between PRMT1 and PRMT8 is a way to recruit PRMT1 to the plasma membrane and locally increase the substrate pool for arginine methylation. PRMT8 and PRMT2 also interact [49], indicating heterodimer formation with PRMT8 may be a family-specific method for directing localization (Fig. 2b). This also indicates that, similar to PRMT1 in trypanosomes [42], PRMT1 in mammals could form a heterotetramer, and this heterotetramer has the potential to include more than one PRMT family member.

Finally, while PRMT7 is not known to homo- or heterodimerize, it does form a pseudo-heterodimer. PRMT7 contains two tandem PRMT domains as a result of genetic duplication [50]. While the N-terminal PRMT domain is well conserved with the canonical PRMT core, the C-terminal PRMT domain exhibits notable substitutions in the SAM- and substrate-binding regions, rendering the C-terminal PRMT7 domain catalytically inactive [51]. Although lacking catalytic activity of its own, the C-terminal domain is required for activity of the N-terminal domain [52] and is important for forming a pseudodimer functional unit with the N-terminal domain that mimics the homodimeric state of other PRMTs [51, 53, 54]. As PRMT9 also contains two tandem PRMT domains, it is suspected that the PRMT9 functional unit may also be a pseudodimer [55]. Compared to other methyltransferases where a separate family member acts as an activator, PRMT7 (and likely PRMT9) is interesting in that a single monomer already contains its activating binding partner in its C-terminal region.

## RNA methyltransferases (METTL3/METTL14)

In contrast to the above methyltransferases, only heterodimerization has been seen among the N6-methyladenosine (m6A) RNA methyltransferases. m6A is an abundant modification found in most eukaryotes that regulates gene expression through its involvement in RNA stability, splicing, and translation [56]. Found in over 30% of RNA transcripts, m6A is an important regulator of neurodevelopment, hematopoiesis, cellular differentiation, and cancer progression [10, 57]. m6A is placed by a number of methyltransferases, including METTL7A, METTL4, METTL16, and METTL5 [58, 59], but the primary and most well-studied m6A methyltransferase is METTL3 [60]. METTL3 is known to be part of a large complex that contains WTAP (Wilms tumor 1-associating protein), ZC3H13 (Zinc finger CCCH-type containing 13), VIRMA (Vir-like m6A methyltransferase associated), RBM15 (RNA binding motif protein 15), HAKAI (E3 ubiquitin-protein ligase), and another member of the METTL family, METTL14 (Fig. 2c) [60–62].

Though initially identified as a second m6A methyltransferase, METTL14 has subsequently been shown to be catalytically inactive on its own [63]. Compared to METTL3, METTL14 possesses an active site variant motif (Fig. 1c), which results in a relatively occluded catalytic site, predicted to prevent the binding of SAM [63–67]. Accordingly, neither SAM nor SAH was found bound in the catalytic site of the METTL14 crystal structure [63, 65, 66]. Similar to DNMT3L, the primary role of METTL14 is to regulate the catalytic activity of METTL3 [63]. The binding of METTL14 to METTL3 to form a heterodimer increases the stability of the complex, and subsequently, activates the catalytic activity of METTL3 [63].

In addition to providing the structural support required for full METTL3 methylation activity, METTL14 is also important for aiding proper RNA substrate binding of the METTL3–METTL14 complex [68]. RNA binding is mediated by both the ZFD of METTL3, and highly basic arginine-glycine repeats (RGG) at the C-terminus of METTL14 [63, 68]. Removal of the METTL14 RGG repeats reduces the ability of METTL14 to bind RNA in vitro and to cross-link with RNA in vivo, indicating these repeats are a second point of RNA contact for the heterodimer [68]. In addition to general RNA binding, METTL14 also contributes to target sequence recognition. A cancer-associated mutant of METTL14, R298P, targets the heterodimer to both canonical 5ʹ-AC-3ʹ methylation sites and aberrant 5ʹ-AU-3ʹ methylation sites, altering the m6A methylation profile and target gene expression [69].

The METTL3–METTL14 interaction is similar to the DNMT3A/B-DNMT3L interaction in that one catalytically inactive “methyltransferase” binds to and activates a separate active family member. Unlike DNMT3A/B, METTL3 is not known to homodimerize or function catalytically without METTL14 [63, 70], and unlike DNMT3L, METTL14 is not known to interact with other METTL family members. However, it will be interesting to see in the future if METTL14 can interact with the other m6A methyltransferases (METTL7A, METTL4, METTL16, and METTL5) in hereto now undiscovered cell-type or cell-cycle dependent manners, as recent evidence (see METTL11A/ METTL11B/METTL13 below) suggests METTL protein complexes can interchange.

## N-terminal methyltransferases (METTL11A/METTL11B/METTL13)

N-terminal protein methylation is a modification that has roles in regulating protein stability, protein–protein, and protein–DNA interactions [71–73]. It can be placed by three different members of the METTL family of methyltransferases, METTL11A, METTL11B, and METTL13 [74–76]. While all three can place the modification, they have very different cellular roles [77]. METTL11A is the primary N-terminal methyltransferase, as it is predicted to trimethylate over 300 substrates based on consensus sequence requirements [74, 78]. METTL11B is a close homolog of METTL11A and shares a conserved MTase catalytic domain (Fig. 1c), but only has monomethylase activity in vitro and no verified in vivo substrates [75]. METTL13 is a dual function methyltransferase that methylates both the N-terminus and lysine 55 (K55) of eukaryotic elongation factor 1 alpha (eEF1A) with a distinct MTase domain for each substrate (Fig. 1c) [76]. Methylation of K55 has been shown to promote translation [6], while the functional role of N-terminal eEF1A methylation remains unknown.

METTL11A and METTL11B have been found to form a heterotrimer consisting of a METTL11A dimer and a METTL11B monomer, and through this interaction, METTL11B stabilizes and subsequently activates the methylation activity of METTL11A against its non-canonical targets [71]. While this was originally suspected to be the result of METTL11B ‘priming’ substrates with monomethylation, the catalytic activity of METTL11B is not required for METTL11A activation [71]. This suggests that METTL11B could primarily act as an inactive regulatory protein, like METTL14, DNMT3L, and PRMT1^PRO^ [77], and instead of priming for METTL11A is promoting a conformational change that favors binding of non-canonical substrates. However, while METTL11B does not currently have any verified in vivo targets, it can bind SAH, it does exhibit in vitro activity, and almost all residues required for substrate binding are conserved between METTL11A and METTL11B [75, 79]. This suggests METTL11B could also be more like PRMT2, with both catalytic and non-catalytic roles. As METTL11A is expressed ubiquitously, and METTL11B displays tissue-specific expression, it may be the non-catalytic, regulatory role of METTL11B is important in certain tissues during times with a high non-canonical substrate burden on METTL11A [71].

METTL11A has also been recently found to participate in an additional regulatory complex with METTL13 [80]. METTL13 is more distantly related to METTL11A than METTL11B [81], and eEF1A is its only known substrate [6, 76]. In contrast to the regulatory effects of METTL11B, METTL13 inhibits the methylation activity of METTL11A [80]. METTL11A also reciprocally regulates METTL13 by inhibiting its N-terminal methylation activity and promoting its K55 methylation activity [80]. The regulatory effects of both METTL11A and METTL13 were found to be independent of their catalytic activities, showing new, non-catalytic, regulatory functions for each [80]. Through its regulatory interaction with METTL13, a new role for METTL11A in translation has been identified [80].

METTL11A is a novel example of methyltransferase regulation through intrafamily heterooligomerization, as it is activated by a potentially inactive family member, but also inactivated by an active family member, who it reciprocally regulates (Fig. 2d). Co-immunoprecipitation data also suggest that all three could interact in one larger complex, and in vitro methyltransferase assays show when all three are present, METTL13 outcompetes METTL11B for regulation of METTL11A [80]. This demonstrates that multiple methyltransferase family members could potentially form regulatory interactions simultaneously (Fig. 2d) and greatly expands the possible methyltransferase regulatory network.

## Methyltransferase interactions in disease

As the myriad of potential methyltransferase regulatory interactions begins to be discovered, it is important to understand how they can be manipulated for treatment of disease. Each of the described methyltransferases plays important biological roles, and accordingly, deviations from their normal functions can result in a variety of human diseases. While it is now commonplace to detect mutations in different diseases and cancers, understanding if a mutation is a driver of disease is still a challenge. First, determining the regulatory interactions of methyltransferases, and second, identifying important residues in the interaction interface, will significantly enhance our ability to understand if and how given mutations are driving protein misregulation and design drugs targeted to this interaction.

**Fig. 3:**
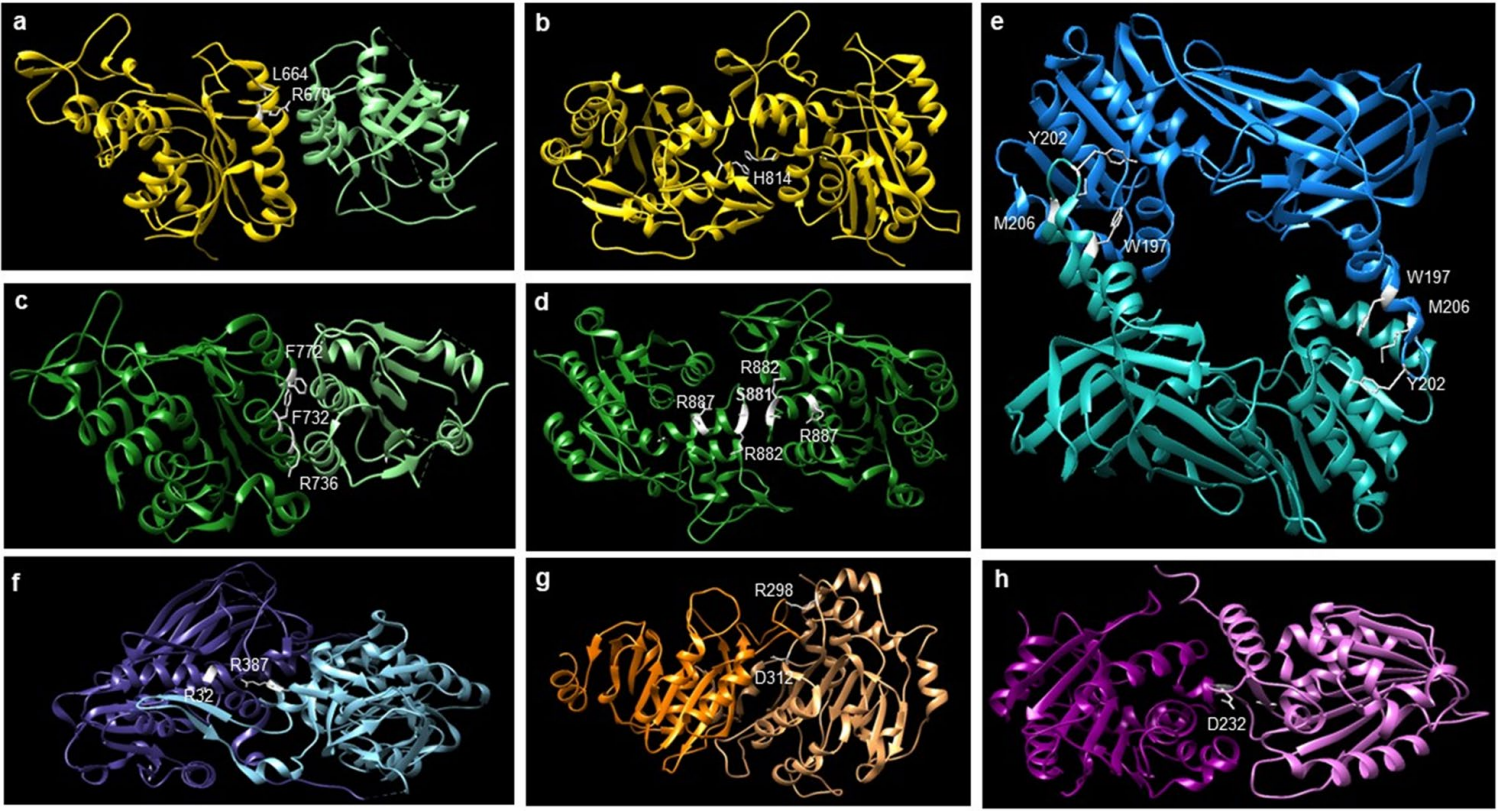
Methyltransferase residues mutated in disease. **a** DNMT3B–DNMT3L (DNMT3B in yellow; DNMT3L in light green) heterodimer with ICF-associated mutated residues in the FF interface shown in white. PDB: 6KDA. **b** DNMT3B–DNMT3B (both subunits in yellow) homodimer with ICF-associated mutated residue in the RD interface shown in white. PDB: 6KDA. **c** DNMT3A–DNMT3L (DNMT3A in dark green; DNMT3L in light green) heterodimer with AML-associated mutated residues in the FF interface shown in white. PDB: 6F57. **d** DNMT3A–DNMT3A (both subunits in dark green) homodimer with AML-associated mutated residues in the RD interface shown in white. PDB: 6F57. **e** Rat PRMT1–PRMT1 (subunits in teal and light teal) homodimer with rat residues orthologous to human cancer-associated mutations shown in white. PDB: 3Q7E. **f** Mouse PRMT7 (NTD in dark blue; CTD in light blue) pseudodimer with mouse residues orthologous to human SBIDDS-associated mutated residues shown in white (amino acid numbering is the same for mouse and human). PDB: 4C4A. **g** METTL3–METTL14 (METTL3 in dark orange; METTL14 in light orange) heterodimer with METTL14 cancer-associated mutated residues shown in white. PDB: 5K7M. **h** METTL11A–METTL11B (METTL11A in purple; METTL11B in pink) heterodimer with METTL11B cancer-associated mutated residue shown in white. PDB: 2EX4; 6DUB; modeled as described previously [80]. All modeled using Chimera UCSF [96]

Several examples of mutations found in human diseases are associated with known protein–protein interactions of methyltransferases (Table 1 and Fig. 3). DNMT3B mutations associated with the FF interface, which mediates the interaction between DNMT3B and either another DNMT3B (in a homotetramer) or DNMT3L (in a heterotetramer), have been found in patients with immunodeficiency, centromeric instability, and facial anomalies (ICF) syndrome (L664P, L664T, R670Q) (Fig. 3a) [27, 82]. All three of these mutations significantly disrupted the DNMT3B–DNMT3B interaction and activity of the DNMT3B–DNMT3B complex, but had differential effects on the DNMT3B–DNMT3L complex [27]. Despite only slight disruptions to the DNMT3B–DNMT3L interaction, L664P/T mutations still showed significant decreases in catalytic activity of the DNMT3B–DNMT3L complex, and R670Q exhibited a slight decrease in activity without significantly disrupting the DNMT3B–DNMT3L interaction [27]. These results show that mutations within the FF interface can have different effects depending on the interaction formed, and that each individual mutation in this region may drive the development of ICF syndrome in different ways [27]. Additionally, there appears to be some interplay between the two interfaces, as the ICF mutation H814R is located at the RD interface (mediates the central DNMT3B–DNMT3B interaction) (Fig. 3b), but disrupts both the RD and the FF interfaces [82].

**Table 1:** Disease-associated mutations in residues near methyltransferase interaction interfaces

Protein	Interface	Mutation	Disease	Notes	Refs.
DNMT3A	RD interface	R882H	AML	Significant decrease in catalytic activity and homooligomerization	[24]
		S881N	AML		[83]
		R887I	AML		[83]
	FF interface	R736H	AML	Destabilized interaction interface, but increased stimulation by DNMT3L	[29]
		F732L	AML		[83]
		F772I	AML		[83]
DNMT3B	FF interface	L664P/T	ICF	Differential effects on activity and disruption of oligomerization	[27]
		R670Q	ICF	Differential effects on activity and disruption of oligomerization	[27]
	RD interface	H814R	ICF	Significant decrease in catalytic activity and oligomerization	[82]
METTL14	METTL3–METTL14	R298P	Endometrial Cancer	Significant decrease in catalytic activity and ability to distinguish substrate from mutant substrate	[63]
		D312Y	Human adult T cell lymphoma/leukemia	Mutation of D312 to A significantly decreased catalytic activity	[63, 83]
PRMT1	PRMT1–PRMT1	W215L	Endometrioid carcinoma-ovary	Significant decrease in catalytic activity, oligomerization, and SAM binding	[84]
		Y220N	Basal cell carcinoma-skin	Significant decrease in catalytic activity, oligomerization, and SAM binding	[84]
		M224V	Adenocarcinoma-colon	Significant decrease in catalytic activity, oligomerization, and SAM binding	[84]
PRMT7	NTD–CTD	R32T	SBIDDS	Residue near NTD–CTD interface in crystal structure	[86]
		R387G	SBIDDS	Residue near NTD–CTD interface in crystal structure	[86]
METTL11B	METTL11A–METTL11B	D232N	Carcinoma-prostate	Significant decrease in METTL11A–METTL11B interaction	[80, 83]

*AML* acute myeloid leukemia, *ICF* immunodeficiency, centromeric instability, and facial anomalies syndrome, *NTD* N-terminal domain, *CTD* C-terminal domain, *SBIDDS* Short Stature, Brachydactyly, Intellectual Developmental Disability, and Seizures syndrome

Similarly, DNMT3A mutations associated with acute myeloid leukemia (AML) can be found at either the RD or FF interface [24, 29, 83]. The second most frequent DNMT3A mutation found in AML, R736H, is found in the FF interface and seems to alter the flexibility of the tetramer and promote methylation (Fig. 3c) [29]. Though the R736H DNMT3A–DNMT3L interface is less stable than the wild type interaction, R736H is more highly activated by DNMT3L, indicating a more flexible interface promotes catalytic activity [29]. Two other AML mutations, F732L and F772I, are found at the DNMT3A–DNMT3L FF interface (Fig. 3c) [83], though their impact on catalytic activity remains to be determined. There are also three AML mutations in DNMT3A found at the RD interface, R882H, S881N, and R887I (Fig. 3d) [24, 83]. While S881N and R887I remain to be studied, it has been shown that R882H alters homooligomerization and decreases the catalytic activity of DNMT3A [24]. In the future, it will be interesting to see if the different DNMT3A mutations differentially affect activity of the homo- and heterotetrameric complexes and promote different disease phenotypes.

In the case of PRMT1, the rat crystal structure has shown that three residues in its homodimerization interface (W197, Y202, and M206) (Fig. 3e) have orthologous residues found mutated in human cancers (W215L, Y220N, and M224V) that disrupt both dimerization and activity [84]. Molecular simulations of W215L, Y220N, and M224V suggest all three lock PRMT1 in a conformation not conducive to dimerization and subsequently disrupt SAM binding and methyltransferase activity [84]. As homo- and heterodimerization interfaces will likely share common residues, it is possible these mutations in the PRMT1 homodimerization interface will also affect the activity of PRMT1–PRMT2, PRMT1-PRMT6, or PRMT1–PRMT8 heterodimers. There are also PRMT7 mutations (R32T and R387G) found in patients with SBIDDS (Short Stature, Brachydactyly, Intellectual Developmental Disability, and Seizures) syndrome [85] that are in the interface between its active N-terminal domain and its inactive C-terminal domain (Fig. 3f) [51, 85, 86]. Interestingly, R32 is in the N-terminal side and R397 is in the C-terminal side [51], indicating mutations in either domain could affect pseudodimerization and subsequent activity of the active N-terminal domain.

For the METTL family, two METTL14 cancer-associated mutations (R298P and D312Y) are located near the METTL3–METTL14 interaction interface [63, 83]. D312 extends directly into the space between the monomers (Fig. 3g), and its mutation has been shown to cause a loss in methylation activity [63]. As mentioned previously, R298P, which is in the highly basic patch near the METTL3–METTL14 junction and close to the catalytic cavity (Fig. 3g), results in a significant loss of methylation activity and the inability of the METTL3–METTL14 complex to distinguish between correct and aberrant methylation motifs [63, 69]. These findings demonstrate how a mutation in a catalytically inactive regulatory methyltransferase can manifest as a disruption to activity of the complex. Additionally, a cancer-associated METTL11B mutant, D232N, has been identified that is at the predicted METTL11A interaction interface (Fig. 3h) and disrupts both the METTL11A–METTL11B interaction [80] and the ability of METTL11B to activate the methylation activity of METTL11A (unpublished data). All three N-terminal methyltransferases are frequently mutated in human cancers [83], which could suggest that METTL11B has its own important catalytic activity, or more similar to METTL14, is important for its regulatory role on METTL11A.

As it is becoming more apparent that targeting protein–protein interactions is a viable option for treatment of disease, drug development is evolving to meet these needs with screening for small molecules or antibodies that disrupt specific interactions and designing peptidomimetic molecules that do the same (reviewed in [87]). Small molecules that disrupt the interaction between MDM2 and p53 are in clinical trials for treatment of AML, metastatic melanoma, and lymphoma [88–90]. A peptide disrupting the same interaction is also in clinical trials for treatment of advanced solid tumors and lymphomas [91]. Antibodies that disrupt the interaction between CD40 and CD40L are in clinical trial for treatment of multiple myeloma, lupus nephritis, and ulcerative colitis [87, 92–94]. Specifically for methyltransferases, small molecule inhibitors targeted to the tetramerization interface of DNMT3A have recently been developed, and one of these inhibitors has promising therapeutic potential, as it is able to induce differentiation of myeloid leukemia cell lines [95]. Further discovery of methyltransferase regulatory interactions will provide even more targets for drug design against protein–protein interactions.

## Conclusions

While heterooligomerization appears to be a shared mode of regulation between close members of the 7βS superfamily, each complex possesses its own unique qualities. A common trend is the regulation of a catalytically active enzyme through binding of an inactive family member, though there are exceptions to this, as seen with PRMT1 and METTL11A. Heterooligomers also tend to have qualities unique from homooligomers of the active enzyme, often enhancing catalytic activity or altering substrate preference. It will be interesting to see if, besides METTL11A and PRMT1, other methyltransferases can form interchangeable complexes or complexes with more than one other family member. As the scope of these interactions has the potential to expand exponentially, more comprehensive mapping is required to better understand the nuances of each regulatory interaction and provide more options for drug design against interaction interfaces.

## Data Availability

Data sharing is not applicable to this article as no datasets were generated or analyzed during the current study.

## Abbreviations

7βS: Seven-beta-strand
ADD: Atrx-Dnmt3-Dnmt3l
AML: Acute myeloid leukemia
CTD: C-terminal domain
DNMT: DNA methyltransferase
eEF1A: Eukaryotic elongation factor 1 alpha
HAKAI: E3 ubiquitin-protein ligase
ICF: Immunodeficiency, centromeric instability and facial anomalies
m6A: *N*6-Methyladenosine
METTL: Methyltransferase-like
MLL: Mixed lineage leukemia
MTase: Methyltransferase
NLS: Nuclear localization signal
NTD: N-terminal domain
PRMT: Protein arginine methyltransferase
PWWP: Pro-Trp-Trp-Pro domain
RBM15: RNA-binding motif-protein RBBP7/RBBP4
SAH: *S*-Adenosyl homocysteine
SAM: *S*-Adenosyl methionine
SAM68: KH domain-containing, RNA-binding, signal transduction-associated protein 1
SBIDDS: Short Stature, Brachydactyly, Intellectual Developmental Disability, and Seizures
SET: Su(var)3–9, Enhancer of zeste, Trithorax
SH3: SRC Homology 3 domain
TIM: Triose-phosphate isomerase
TRP: Tetratricopeptide
VIRMA: Vir-like m6A methyltransferase associated
WTAP: Wilms’ tumor-1(WT1)-associating protein
ZC3HI3: Zinc finger CCCH domain-containing protein 13
ZFD: Zinc finger domain

## Glossary

DNA methyltransferase (DNMT) family: Enzyme family that is part of the seven-β-strand superfamily. Members place a methyl group on an aromatic base contained in a DNA nucleotide
FF interface: Interface between DNMT3 monomers
Heterodimerization: Interaction of two different proteins
Heterooligomerization: Interaction of two or more different proteins
Homodimerization: Interaction of two of the same protein
Homooligomerization: Interaction of two or more of the same protein
Methyltransferase: Enzyme that catalyzes the transfer of a methyl group from the methyl donor *S*-adenosyl-methionine
Methyltransferase-like (METTL) family: Enzyme family that is part of the seven-β-strand superfamily. Members can place methyl groups on DNA, RNA, and proteins
Protein arginine methyltransferase (PRMT) family: Enzyme family that is part of the seven-β-strand superfamily. Members place methyl groups on arginine side chains
RD interface: Interface between DNMT3 dimers
*S*-adenosyl-methionine (SAM): Methyl group donor and cofactor utilized by methyltransferases
*S*-adenosyl-homocysteine (SAH): Analog of SAM that cannot be metabolized by methyltransferases and is, therefore, a methyltransferase inhibitor
Seven-β-strand (7βS) superfamily: Group of combined methyltransferase families that share a conserved SAM binding domain that is formed by part of a seven-β-strand structure
Superfamily: Grouping of structurally similar methyltransferase families
